# Therapeutic efficacy of mesenchymal stem cells in periodontal regeneration: a systematic review and meta-analysis of clinical trials

**DOI:** 10.12701/jyms.2026.43.42

**Published:** 2026-06-24

**Authors:** Fiki Muhammad Ridho, Anis Irmawati, Raed Labib, Hayyu Norma Almira, Ridwan Alfatah, Bayu Cahyo Bintoro

**Affiliations:** 1 Department of Dental Medicine, Faculty of Dentistry, Universitas Airlangga, Surabaya, Indonesia; 2 Department of Basic Dental Science and Public Health, Faculty of Dentistry, Universitas Airlangga, Surabaya, Indonesia; 3 Department of Oral Surgery, Faculty of Dentistry, 21 September University for Medical and Applied Sciences, Sana’a, Yemen; 4 Department of Medicine, Faculty of Medicine, Universitas Islam Negeri Walisongo, Semarang, Indonesia

**Keywords:** Dentistry, Mesenchymal stem cells, Periodontal regeneration, Periodontitis, Tissue engineering

## Abstract

**Background:**

Periodontitis is a major global health problem that leads to destruction and loss of periodontal tissues. As conventional therapy has a limited capacity to restore the complex architecture of the periodontium, regenerative approaches have been explored. This meta-analysis evaluates the therapeutic efficacy of mesenchymal stem cells (MSCs) for periodontal regeneration in patients with periodontal defects.

**Methods:**

PubMed, Scopus, Web of Science, Dentistry & Oral Sciences Source, and the Cochrane Library were searched for clinical trials. The risk of bias was assessed using Cochrane RoB2. Random- or fixed-effects meta-analyses were used in the pooled analysis and reported as mean differences (MDs) and 95% confidence intervals (CIs). Certainty of evidence was rated using Grading of Recommendations, Assessment, Development, and Evaluation (GRADE).

**Results:**

Ten clinical trials were included in the analysis. MSCs significantly improved probing pocket depth (MD, –0.51; 95% CI, –0.84 to –0.19; *p*<0.01; *I*^2^=62%), clinical attachment level (MD, –0.75; 95% CI, –0.95 to –0.54; *p*<0.01; *I*^2^=40%), cementoenamel junction to the base of defect (MD, –0.49; 95% CI, –0.95 to –0.02; *p*=0.04; *I*^2^=63%), and intrabony defect (MD, –1.09; 95% CI, –1.60 to –0.57; *p*<0.01; *I*^2^=71%). However, no significant improvement in gingival recession was observed (MD, –0.18; 95% CI, –0.38 to 0.01; *p*=0.07; *I*^2^=0%). The GRADE assessment indicated a high-to-moderate certainty.

**Conclusion:**

MSCs represent a promising strategy for periodontal regeneration. Nevertheless, the limited number of studies and the observed heterogeneity warrant cautious interpretation. Larger methodologically rigorous trials are needed to clarify the true contribution of MSCs to periodontal regeneration.

## Introduction

Periodontitis is a chronic inflammatory condition involving the periodontium, which comprises the supporting structures of the teeth. This disease has a high burden, as evidenced by more than one billion people with severe periodontitis in 2021, and this number is projected to continue increasing over the coming decades [1]. Although this disease is often overlooked, several studies have revealed that it can impair quality of life and increase the risk of various adverse systemic diseases [2-7]. Furthermore, chronic inflammatory and immune responses in severe periodontitis result in severe destruction of the alveolar bone and loss of periodontal tissues. If left untreated, this gradually diminishes alveolar bone height and weakens the periodontium, ultimately leading to tooth loss [8-10].

Scaling and root planing, a conventional nonsurgical periodontal therapy, is the initial therapy that primarily aims to control microbial infections and suppress the inflammatory burden associated with periodontitis [11]. Although this strategy can improve the clinical attachment level (CAL) and probing pocket depth (PPD), as well as help stabilize disease progression, its therapeutic impact is largely limited to disease control rather than genuine regeneration of the affected periodontal tissues [12,13]. Specifically, conventional treatment often fails to restore the complex periodontal architecture, resulting in incomplete structural and functional recovery [14,15]. In addition, the clinical outcomes after conventional therapy remain highly variable and are influenced by factors such as defect morphology, disease severity, individual patient response, and lifestyle status [16]. Collectively, these limitations highlight the need for adjunctive therapeutic approaches to address the inherent regenerative deficits in advanced periodontitis.

Regenerative strategies based on tissue engineering principles have emerged as promising options for the management of advanced periodontal defects. Tissue engineering aims to regenerate lost periodontium to restore tissue function through coordinated interactions among progenitor cells, scaffolds or supportive matrices, and bioactive signaling molecules [17,18]. Among the cellular components explored, mesenchymal stem cells (MSCs) have attracted substantial attention due to their multipotent differentiation capacity and immunological properties, including anti-inflammatory, immunoregulatory, immunosuppressive, and wound-healing effects to promote tissue repair [19-21]. MSCs can be isolated from multiple sources, including adipose tissue, bone marrow, and dental and periodontal tissues; each MSC type has distinct biological characteristics and regenerative potential [22].

Several previous meta-analyses have evaluated the effects of MSC-based therapies on periodontal regeneration in clinical trials. However, most have indicated substantial heterogeneity and have not performed adequate subgroup analyses to identify their sources, including analyses stratified by MSC type or origin. These limitations hinder the formulation of precise clinical conclusions regarding the relative effectiveness of the various MSC approaches. Therefore, this meta-analysis aimed to synthesize clinical evidence on the efficacy of MSCs in periodontal regeneration and perform subgroup analyses based on study design, MSC type, and follow-up duration, with the aim of reducing heterogeneity and enhancing the clinical applicability of the findings.

## Methods

**Ethics statement:** Ethical approval was not required because this study was conducted as a systematic review and meta-analysis based on previously published studies.

### 1. Protocol and registration

This study was conducted as a systematic review and meta-analysis following the Preferred Reporting Items for Systematic reviews and Meta-Analyses guidelines [23]. The review protocol was registered in the PROSPERO (International Prospective Register of Systematic Reviews) under registration number CRD420261369384.

### 2. Eligibility criteria

The inclusion criteria for the clinical trials were: (1) randomized controlled trials (RCTs) involving a treatment group receiving MSC-based therapy and a control group receiving another treatment strategy without stem cells; (2) trials involving systemically healthy adult participants with stage III–IV/moderate-severe periodontitis accompanied by periodontal defects; (3) studies with patients who had undergone at least phase I periodontal therapy (nonsurgical) before receiving MSC-based treatment or other cell-free treatment strategies; (4) studies that reported outcomes including PPD, defined as the distance between the gingival margin and the base of periodontal pocket; CAL, defined as the distance from the cementoenamel junction (CEJ) to the base of periodontal pocket; gingival recession (GR), defined as the distance from the CEJ to the gingival margin; CEJ-BD, defined as the distance from the CEJ to the base of defect; and intrabony defect (IBD), defined as the distance from the alveolar bone crest to the base of the intrabony defect; and (5) RCTs published in English-language peer-reviewed journals.

RCTs were excluded if they employed a pre-post intervention design without a control group, failed to provide complete outcome data, presented results solely in the form of figures/diagrams without numerical data, or were categorized as preclinical/animal studies, reviews, or case studies/reports/series.

### 3. Information sources and search strategy

PubMed, Scopus, Web of Science, Dentistry & Oral Sciences Source, and the Cochrane Library were searched electronically. Backward and forward citation searches were also performed to ensure that no relevant clinical trials were missed during identification. Gray literature was not searched because a reproducible search strategy could not be clearly specified for such sources, and many of these records had not undergone peer review [24]. This comprehensive search was conducted in October 2025 and repeated in March 2026 by two investigators (FMR and AI). The search was limited to articles published in peer-reviewed journals and written in English. No restrictions were applied regarding the publication year to ensure the comprehensive retrieval of relevant evidence.

The two investigators (FMR and AI) combined several keywords using Boolean operators (AND, OR) in this search, including “stem cells,” “mesenchymal stem cells,” “MSC,” “mesenchymal stromal cells,” “periodontal,” “periodontitis,” “periodontal disease,” “periodontal regeneration,” “randomized controlled trial,” “RCT,” and “clinical trial.” The full search string used for PubMed was: ((“stem cells”[MeSH Terms] OR “stem cells”[All Fields]) OR (“mesenchymal stem cells”[MeSH Terms] OR “mesenchymal stem cells”[All Fields] OR “MSC”[All Fields]) OR (“mesenchymal stromal cell”[All Fields])) AND ((“periodontal”[All Fields] OR “periodontally”[All Fields] OR “periodontically”[All Fields] OR “periodontics”[MeSH Terms] OR “periodontics”[All Fields] OR “periodontic”[All Fields] OR “periodontitis”[MeSH Terms] OR “periodontitis”[All Fields] OR “periodontitides”[All Fields])) AND (“randomized controlled trial” OR “RCT” OR “clinical trial” OR “controlled clinical trial” OR “random” OR “trial” OR “placebo”) NOT (“review” OR “narrative review” OR “literature review” OR “scoping review” OR “rapid review” OR “umbrella review” OR “integrative review” OR “critical review” OR “systematic review” OR “meta-analysis” OR “case study” OR “case report” OR “case series” OR “commentary” OR “editorial” OR “short communication” OR “animal study” OR “animal” OR “in vivo” OR “mouse” OR “mice” OR “murine”). A detailed search strategy for all databases is presented in Supplementary Table 1.

### 4. Selection process

Raw files in RIS and BibTeX formats from each database were imported into Zotero 9.0 for Windows (Corporation for Digital Scholarship, Vienna, VA, USA). This process automatically removed all duplicates. Articles were initially screened based on the title and abstract, and those irrelevant to the research topic were excluded. Articles deemed relevant were subsequently assessed for eligibility by applying the previously defined eligibility criteria. Articles excluded at this stage were reported together with the specific reasons for exclusion. Two researchers (FMR, RL) conducted this process with involvement of the supervisor (AI) in cases of discrepancy.

### 5. Data collection process

Two authors (RL, RA) independently extracted data from the included studies by reading the full texts and documenting them in Microsoft Excel 2016 for Windows (Microsoft Corp., Redmond, WA, USA). Several key data elements such as authors, study design, participants, age, periodontal status, intervention, control, study duration, and adverse event assessment were collected. For the data required for quantitative synthesis, the outcome data at baseline and follow-up time-points for PPD, CAL, GR, CEJ-BD, and IBD outcomes were extracted and reported as means and standard deviations (SDs). If data were reported as medians, they were converted to means and SDs when possible, using the model from Wan et al. [25]. In studies with multiple follow-up time-points or doses, all reports across all follow-up time-points and doses were extracted. The extracted data were validated by a third investigator (FMR) and disagreements were resolved through discussion.

### 6. Risk of bias assessment

Risk of bias (RoB) was evaluated using the Cochrane RoB2 tool [26]. Two assessors (FMR, RL), who had previously calibrated their interpretation of the signaling questions using the RoB tool, independently performed this assessment. The senior author (AI) was involved in the deliberation if discrepancies arose between the two reviewers during the evaluation process. The RobVis tool [27] was used to visualize the assessment results.

### 7. Statistical analysis

The data used in the pooled analysis were derived from each outcome expressed as means and SDs, by comparing the results of the MSC intervention group and the cell-free control group. Random- or fixed-effects analyses were used to pool the effect sizes across studies. The results are expressed as mean differences (MDs) with 95% confidence intervals (CIs). A *p*-value <0.05 was considered statistically significant.

To assess heterogeneity, the chi-square test was used, with a *p*-value <0.10 indicating the presence of heterogeneity, along with the *I*² statistic, with value categories of 0% to 25% (low), 25% to 50% (moderate), and 50% to 100% (high) [28]. The meta-analysis model applied was a random-effects model using the restricted maximum likelihood method when *I*²>50%; otherwise, a fixed-effects model using the inverse-variance method was used [29].

Subgroup meta-analysis was conducted based on study design (split-mouth vs. parallel-arm), type of MSC (orally derived MSCs vs. non-orally derived MSCs), and follow-up duration (≤6 vs. >6 months). The leave-one-out method was used for sensitivity analysis. Statistical analyses were performed using StataMP 17.0 for Windows (StataCorp LLC., College Station, TX, USA).

### 8. Reporting bias assessment

Funnel plots, which plot MDs against standard errors, were used to evaluate publication bias. This assessment was performed only if an outcome included at least 10 studies in the analysis [30]. The funnel plots were visually interpreted by two investigators (FMR, AI). Furthermore, formal analyses using Egger’s test [31] and Begg’s test [32] were performed to statistically confirm publication bias, with *p*<0.10 indicating the presence of bias.

### 9. Certainty of evidence

Two investigators (AI, RL) evaluated the evidence certainty using Grading of Recommendations, Assessment, Development, and Evaluation (GRADE) [33]. This approach assesses several domains, including RoB, inconsistency, indirectness, imprecision, and publication bias. Three levels of judgment were applied: not serious, serious, and very serious. Downgrading of the level of evidence was performed based on the assessment of each domain, with no downgrade for a judgment of “not serious,” one level of downgrade for “serious,” and two levels of downgrade for “very serious.”

## Results

### 1. Study selection

After the exclusion of 175 duplicates, 296 records were identified through electronic database searching. Due to their irrelevance to this study, 264 records were excluded during the initial screening, leaving 32 studies. After eligibility was assessed through a full-text review, 23 full-text articles were excluded for specific reasons, including case reports/series, in vitro studies, animal studies, irrelevant aims, qualitative studies, ineligible populations, ineligible interventions, ineligible controls, undefined outcomes, and unavailable data, resulting in nine included studies. In the citation search, 12 records were identified; however, 11 were excluded, leaving one eligible study. Overall, 10 clinical trials [34-43] were finally included (Fig. 1).

### 2. Study characteristics

Ten RCTs involving 276 patients (108 patients/teeth/sites in the treatment group and 151 patients/teeth/sites in the control group) aged ≥18 years with periodontitis and intrabony defects were included in this study. Three RCTs [36,38,42] used a split-mouth design, six RCTs [34,35,39-41,43] used a parallel-arm design, and one RCT [37] used teeth as the unit. Based on the type of MSC administered, six RCTs used orally derived MSCs, including gingival MSCs [34], periodontal ligament MSCs [36,37,41], and dental pulp MSCs [39,40]. Four other RCTs administered non-orally derived MSCs, including bone marrow MSCs [35], umbilical cord MSCs [38], peripheral blood MSCs [42], and adipose MSCs [43]. Regarding study duration, four studies [34,38,40,42] followed up for 6 months, two studies [36,43] for 9 months, and four RCTs [35,37,39,41] for 12 months (Table 1).

### 3. Risk of bias in studies

The RoB assessment indicated that five trials [34,39-42] were judged as having a low RoB, while the remaining five trials [35-38,43] were judged as having some concerns (Fig. 2). Domain 4 was most frequently judged as having concerns because several RCTs did not clearly report the blinding of the examiners in the outcome measurements. Some RCTs were also judged as having concerns in Domain 1, where several studies failed to describe the randomization process or allocation concealment. Finally, no studies or domains were judged to have a high RoB.

### 4. Meta-analysis

The random-effects analysis showed that compared with the control, MSCs significantly reduced PPD (MD, –0.51; 95% CI, –0.84 to –0.19; *p*<0.01); however, considerable heterogeneity was found (*I*²=62%, *p*<0.01) (Fig. 3A). A noteworthy gain in CAL was also observed in the MSC group based on fixed-effects analysis (MD, –0.75; 95% CI, –0.95 to –0.54; *p*<0.01), with moderate heterogeneity (*I*²=40%, *p*=0.03) (Fig. 3B). Nevertheless, fixed-effects analysis showed that MSCs did not significantly improve GR (MD, –0.18; 95% CI, –0.38 to 0.01; *p*=0.07) (Fig. 3C).

The random-effects meta-analysis also showed that MSCs significantly improved CEJ-BD (MD, –0.49; 95% CI, –0.95 to –0.02; *p*=0.04) with high heterogeneity (*I*²=63%, *p*=0.02) (Fig. 4A) and improved IBD (MD, –1.09; 95% CI, –1.60 to –0.57; *p*<0.01) with substantial heterogeneity (*I*²=71%, *p*<0.01) (Fig. 4B).

### 5. Subgroup analysis

To explore the moderate heterogeneity observed for CAL outcomes (*I*²=40%) and the substantial heterogeneity observed for PPD (*I*²=62%), CEJ-BD (*I*²=63%), and IBD (*I*²=71%) outcomes, subgroup analysis was performed by stratifying the studies according to study design (split-mouth vs. parallel-arm), MSC type (orally derived vs. non-orally derived), and follow-up duration (≤6 vs. >6 months) (Table 2). For the study design subgroup analysis, the study by Chen et al. [37] was excluded because it used teeth as the unit of analysis and its design was insufficiently clear to be classified as either split-mouth or parallel-arm. Furthermore, this was the only study that employed such a design/unit structure, precluding meaningful subgroup comparisons.

For the PPD outcome, the effect estimate and subgroup-between significance did not change substantially when stratified by study design (*p*=0.52), MSC type (*p*=0.96), or follow-up duration (*p*=0.92); however, heterogeneity decreased markedly to low and nonsignificant levels in studies using a split-mouth design (*I*²=0%, *p*=0.74), non-orally derived MSCs (*I*²=0%, *p*=0.82), and in trials with follow-up durations of >6 months (*I*²=1%, *p*=0.51). These findings indicate that the study design, MSC type, and follow-up duration are likely contributing factors to the substantial heterogeneity in PPD outcomes.

For the CAL outcome, the effect estimates and significance also remained unchanged, and the differences between subgroups were not significant after stratification by study design (*p*=0.47), MSC type (*p*=0.57), or follow-up duration (*p*=0.35). In addition, the *I*² statistic indicated moderate heterogeneity across all stratifications, suggesting that the study design, MSC type, and follow-up duration did not explain the moderate heterogeneity in CAL outcomes.

For the CEJ-BD outcome, heterogeneity was low (*I*²=0%, *p*>0.10) after stratification by study design and MSC type; however, the effect in studies using a split-mouth design and orally derived MSCs became nonsignificant, whereas the magnitude of the effect in studies using a parallel-arm design and non-orally derived MSCs increased and became significant between the two subgroups (*p*<0.01). In contrast, stratification by follow-up duration yielded a nonsignificant effect estimate, with heterogeneity remaining high. However, it is crucial to note that only a limited number of studies have reported the CEJ-BD outcome.

Finally, for the IBD outcomes, stratification by study design did not show significant between-subgroup differences (*p*=0.26). The split-mouth subgroup showed a nonsignificant and imprecise pooled effect, whereas the parallel-arm subgroup showed a significant effect. However, decreased heterogeneity was observed only in studies with a split-mouth design (*I*²=47%, *p*=0.17). The analysis further showed that studies using non-orally derived MSCs produced a significant effect, with a reduction in heterogeneity to a nonsignificant level (*I*²=28%, *p*=0.14), whereas the effect in studies using orally derived MSCs became nonsignificant and heterogeneity remained high. Meanwhile, based on follow-up duration, both studies with ≤6 and >6 months of follow-up showed significant effects, but heterogeneity decreased only in studies with follow-up durations of >6 months (*I*²=34%, *p*=0.21). This indicates that study design, MSC type, and follow-up duration may also contribute to the high heterogeneity observed in IBD outcomes.

### 6. Sensitivity analysis

The analysis showed that the effect estimates and significance for the PPD (Fig. 5A), CAL (Fig. 5B), and IBD (Fig. 5E) outcomes remained stable after the sequential exclusion of individual studies and reanalysis of the remaining articles. Meanwhile, for the GR outcome (Fig. 5C), the MD values consistently showed a negative direction of effect with relatively small changes. However, the results tended to be inconsistent, indicating that the effect for the GR outcome was less robust. For the CEJ-BD outcome (Fig. 5D), the direction of effect also remained negative, but the observed significance fluctuated more, indicating that the effect estimate for CEJ-BD was sensitive to the exclusion of individual studies and was not yet fully stable. The results from the sensitivity analysis suggest that the interpretation of the findings requires caution.

### 7. Publication bias

Publication bias analysis was performed for outcomes involving at least 10 studies. Visual inspection of the funnel plots for PPD (Fig. 6A) and CAL (Fig. 6B) showed a tendency toward symmetry. Furthermore, formal analyses using Egger’s test (*p*=0.63 for PPD, *p*=0.87 for CAL) and Begg’s test (*p*=0.83 for PPD, *p*=0.50 for CAL) detected no publication bias.

### 8. Assessment of adverse events

All included studies reported adverse event assessments. Overall, all studies indicated that the MSC intervention appeared to be safe and well tolerated (Table 3). More specifically, seven of the 10 included studies reported no adverse effects or complications during the study period [34-36,38,39,41,42]. Although Chen et al. [37] did not report any major adverse effects and blood and serum parameters remained within normal limits, they noted swelling and moderate pain, and two patients had positive albumin findings in urine tests. In addition, Liu et al. [40] reported that some patients experienced toothache and injection-related swelling, as well as gingival swelling and diarrhea, which may have been associated with the injection. In the study by Tobita et al. [43], two patients experienced episodes of acute periodontitis and heart failure, respectively; however, both events were considered unrelated to MSC administration.

### 9. Certainty of evidence

Based on the GRADE evaluation, a high level of evidence was found for CAL outcomes and a moderate level of evidence for PPD, GR, CEJ-BD, and IBD outcomes (Table 4). The primary reasons for downgrading were related to the inconsistency domain due to substantial *I*² values (PPD, CEJ-BD, and IBD) and the imprecision domain due to the CI crossing the line of no significance (GR).

## Discussion

This meta-analysis evaluated the therapeutic efficacy of MSCs for periodontal regeneration in patients with periodontal defects. Overall, the findings indicated that MSC-based therapy was associated with significant improvements in PPD, CAL, CEJ-BD, and IBD, whereas no significant effect was observed for GR. However, from a clinical perspective, the magnitudes of these effects should be interpreted with caution. Although the pooled estimates suggested a favorable regenerative signal, several absolute differences were modest, and their practical relevance may depend on baseline defect morphology, disease severity, MSC type, and the extent to which MSCs were delivered with adjunctive biomaterials. Sensitivity analyses showed that the PPD, CAL, and IBD outcomes were relatively robust, whereas the effects on GR and CEJ-BD were more susceptible to the influence of certain studies. Although no publication bias was identified for the analyzable outcomes and the level of evidence ranged from moderate to high, caution is warranted for outcomes characterized by substantial heterogeneity and a limited evidence base.

Moderate-to-substantial heterogeneity was observed mainly in the PPD, CAL, CEJ-BD, and IBD outcomes. Exploratory subgroup analyses suggested that non-orally derived MSCs were associated with more consistent improvements in these outcomes and generally lower levels of heterogeneity. However, this finding should not be interpreted as evidence of clear source superiority because the number of studies in each subgroup was small, and the analyses were exploratory. In contrast, subgrouping by study design did not consistently explain the heterogeneity observed for PPD and CAL, as both split-mouth and parallel-arm studies generally showed significant effects without significant between-subgroup differences. For CEJ-BD and IBD, the apparent differences between split-mouth and parallel-arm studies should be interpreted cautiously because only a limited number of studies were available for each subgroup, particularly for CEJ-BD. Furthermore, some reductions in heterogeneity were observed within specific design subgroups; however, these findings were not sufficiently robust to indicate that the study design was a major source of heterogeneity. These findings may also reflect methodological differences in analytical units, because some trials analyzed outcomes at the patient level [34,35,39-41,43], whereas others used site- [36,38,42] or tooth-based units [37], which may introduce dependencies and affect variance estimation. In addition, the remaining heterogeneity may have been influenced by substantial variations in control conditions, scaffold or adjunct biomaterials, cell dose, delivery methods, and baseline periodontal defect characteristics across the trials. Collectively, such variability likely contributed to residual heterogeneity and limited direct comparisons across studies.

Several findings of this meta-analysis agree with previous meta-analyses [44,45], particularly for the PPD, CAL, GR, and IBD outcomes. Importantly, the present study extends the existing evidence base by incorporating the most recent RCTs and performing subgroup analyses according to study design, MSC type, and follow-up duration. Regarding CEJ-BD outcomes, this study showed a significant effect, which was different from that reported by Sun et al. [45]. This difference likely reflects an expanded evidence base that includes more recent studies. Consistent with this, Campagna et al. [46], who specifically focused on orally derived MSCs, reported no significant differences in the improvement in PPD, CAL, GR, or radiographic bone gain compared to controls. In contrast, our subgroup analysis of orally derived MSCs showed significant improvements in PPD and CAL. Nevertheless, these findings should be interpreted conservatively, because the subgroup analyses were exploratory and based on relatively few studies.

Biologically, the regenerative effects of MSCs are thought to be mediated mainly by paracrine mechanisms, through which MSCs can respond to an inflammatory microenvironment and induce a more pro-reparative milieu [47,48]. In response to inflammatory cytokines, MSCs release anti-inflammatory factors and immunomodulatory mediators that stimulate tissue repair and induce macrophage polarization toward the M2 phenotype [49-51]. Furthermore, MSCs exert both direct and indirect effects to control oxidative stress and inflammatory processes by reducing elevated reactive oxygen species and cytokine levels, which are characteristic features of periodontitis [52]. In addition, MSCs secrete various bioactive factors including pleiotropic cytokines, chemokines, and growth factors, which enhance the regenerative environment and subsequently promote periodontal repair [53,54]. Considering that the destruction of periodontal tissue is largely driven by persistent chronic inflammation, these mechanisms provide a biologically plausible explanation for the regenerative benefits of MSC therapy.

Vascularization is crucial for successful bone and tissue regeneration because it provides adequate nutrients, growth factors, minerals, and oxygen for recovery [55,56]. Through various secretomes, MSCs contribute to angiogenesis, endothelial progenitor cell migration, and antiapoptotic processes [57,58]. Angiogenesis is essential for tissue regeneration because it prevents ischemia at the healing site, and the formation of new vessels provides signaling molecules involved in osteogenesis and cell differentiation [59,60]. Taken together, this evidence suggests that the clinical benefits of MSCs extend beyond direct cell replacement and are likely attributable to coordinated immunomodulatory, angiogenic, and regenerative effects.

The safety profile of MSCs appears favorable, as most RCTs indicated no serious adverse events and only mild, self-limiting complaints such as swelling, transient pain, toothache, or gastrointestinal symptoms [34-43]. This finding supports the notion that MSC therapy is generally well tolerated and does not introduce major safety concerns in the clinical setting. Indeed, previous studies have demonstrated that the clinical use of MSCs yielded positive outcomes with a favorable safety profile and without any serious adverse effects [61-63]. Nevertheless, the possibility of complications related to MSC-based therapy should be considered and evaluated over the long term as MSC intervention remains a potentially risky therapy [64]. It is important to note that the available safety data should still be interpreted cautiously because the evidence is derived from relatively small study populations and several trials had short follow-up periods, which may have led to an underestimation of minor or delayed complications. Furthermore, some reported events cannot be confidently attributed to MSC administration, because they may also reflect the underlying disease process or concomitant procedural intervention. Therefore, firm conclusions regarding the safety profile of MSCs for periodontal regeneration require larger studies with standardized safety reporting and longer follow-up periods to better capture potentially delayed adverse events.

The study findings indicate that MSC-based therapy has the potential to serve as a promising regenerative adjunct in patients with periodontitis and intrabony defects, particularly for improving periodontal clinical and radiographic parameters (PPD, CAL, CEJ-BD, and IBD), although its effect on GR has not been consistent. The subgroup analysis also suggests that the clinical response is influenced by the study design, MSC type, and follow-up duration; however, this should be interpreted as hypothesis-generating rather than definitive. In practice, the choice of cell source, delivery strategy, and accompanying biomaterials should be carefully considered. However, given that the interstudy heterogeneity remains substantial and a limited number of studies have been reported for several outcomes, the clinical application of MSCs still requires protocol standardization, further clinical trials with larger samples, and long-term evaluation before it can be broadly recommended as a routine therapy for periodontal regeneration.

This study has several limitations. First, the number of eligible trials was small for some outcomes, which constrained the robustness of the pooled estimates. Second, substantial overall heterogeneity persisted in several findings and the subgroup analyses were exploratory, with some strata consisting of only a small number of studies. Third, the included studies were not fully homogeneous with respect to participants’ baseline periodontal conditions, intervention composition, control conditions, use of scaffolds or biomaterials, cell doses, MSC preparation protocols, and follow-up duration, which likely contributed to residual heterogeneity. Fourth, as several trials have combined MSCs with adjunctive biomaterials or regenerative procedures, the independent contribution of MSCs alone cannot be fully isolated from the effect of the overall treatment complex. Fifth, assessment of publication bias was feasible only for PPD and CAL because the remaining outcomes were supported by too few studies, limiting a comprehensive evaluation. Sixth, the search was restricted to peer-reviewed journal articles, and gray literature sources were not included; therefore, potentially relevant unpublished or non-peer-reviewed studies may have been missed.

Building on the limitations identified in this study, multicenter RCTs with larger sample sizes are needed, particularly for outcomes currently supported by a limited number of studies. Study designs should also be more standardized, from baseline periodontal criteria, defect characteristics, analytical units, and the type and dose of MSCs, to the composition of biomaterials and the control conditions used, so that between-study heterogeneity can be reduced and effect estimates become more robust. In addition, clinical trials with longer follow-up durations are necessary to assess the stability of regenerative outcomes and evaluate adverse events. Thus, evidence of the actual contribution of MSCs can be obtained with a higher level of certainty.

We conclude that MSCs have considerable potential for periodontal regeneration in patients with periodontal defects. However, given the relatively limited number of studies available for some outcomes and the high overall heterogeneity, the findings should be interpreted with caution. Future clinical trials involving larger samples and meticulous methodologies are required to confirm the current meta-analytic evidence.

## Figures and Tables

**Fig. 1.**
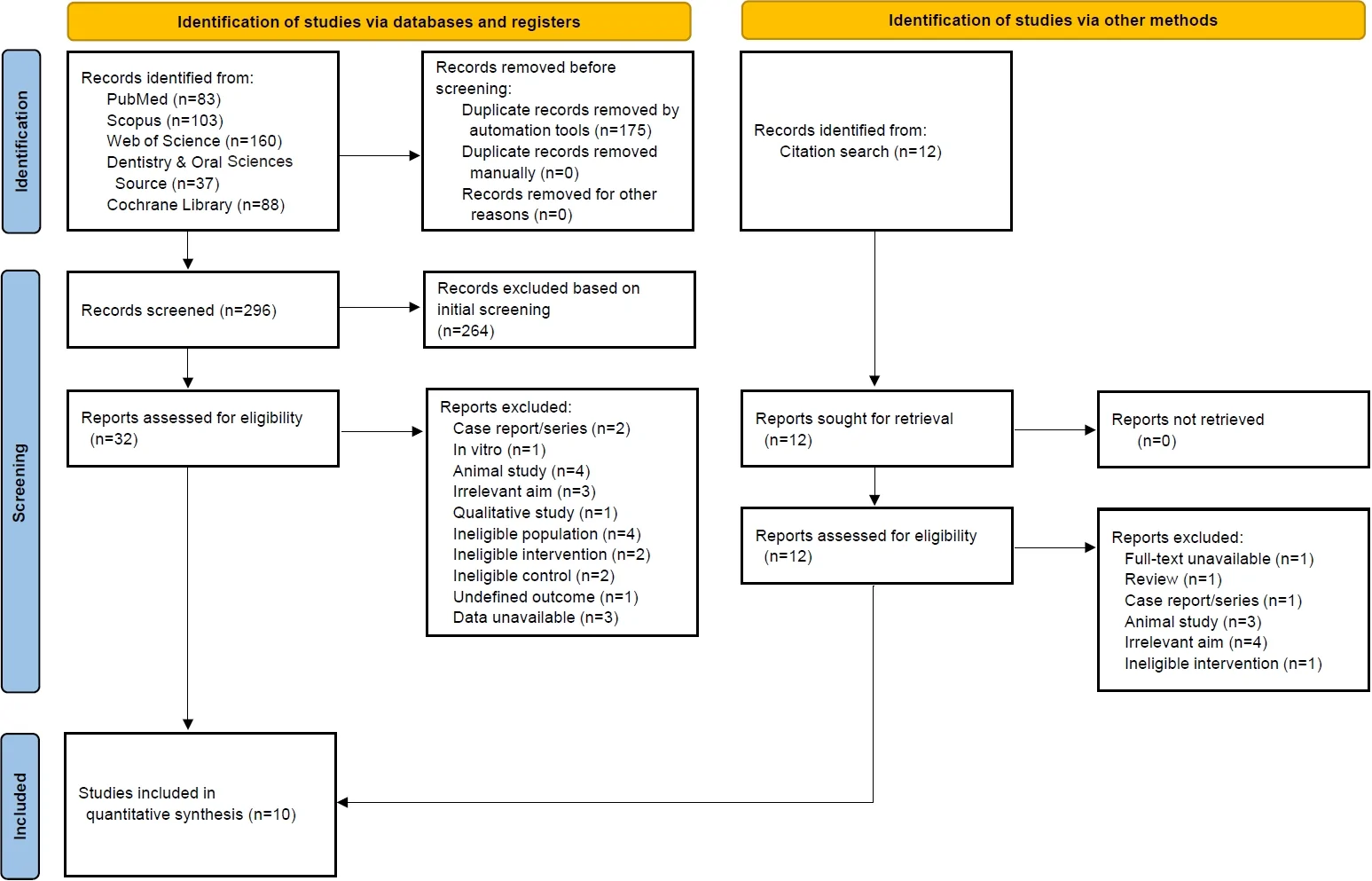
Preferred Reporting Items for Systematic reviews and Meta-Analyses flowchart.

**Fig. 2.**
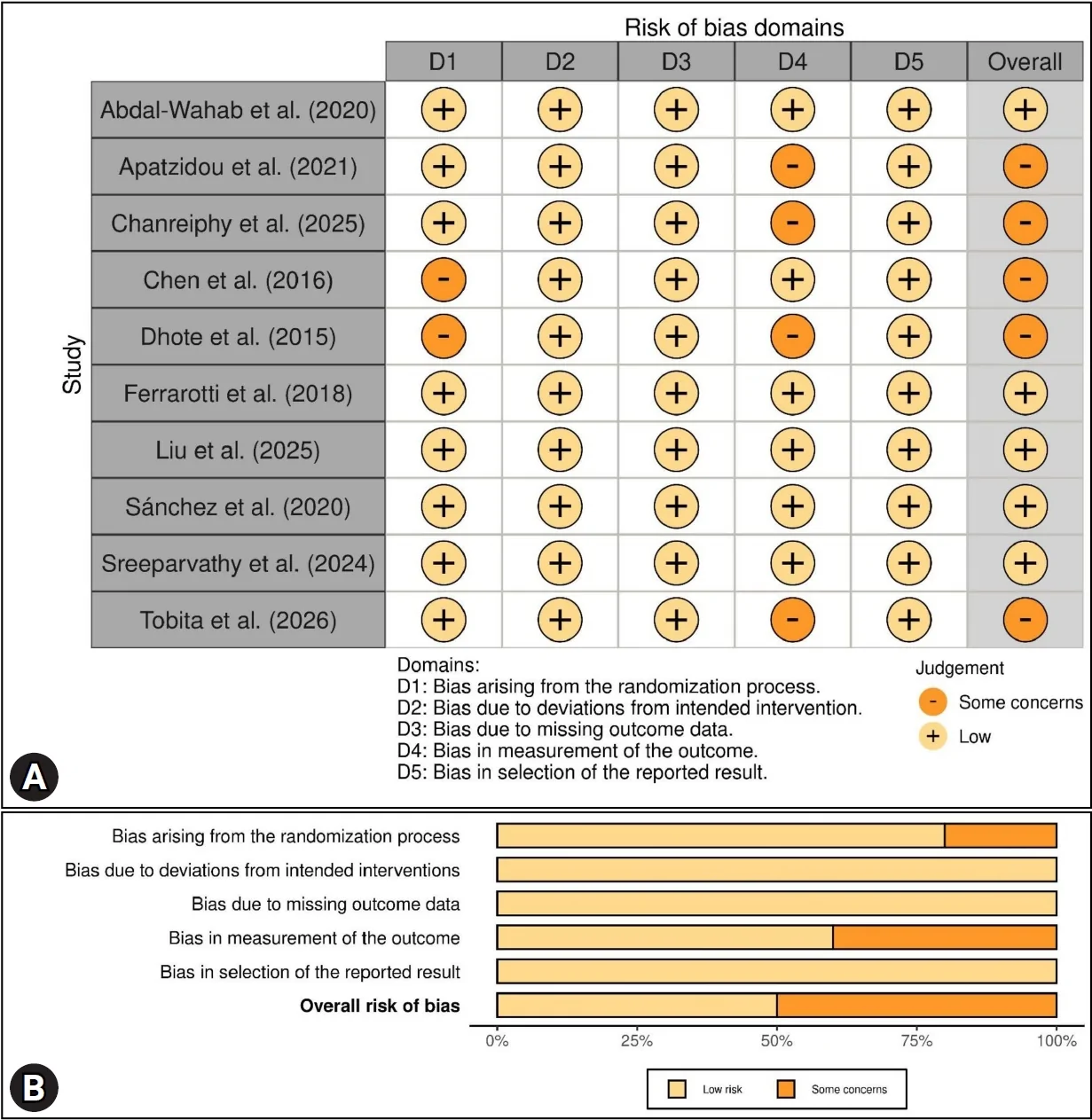
Risk of bias assessment using RoB2: (A) summary and (B) graph.

**Fig. 3.**
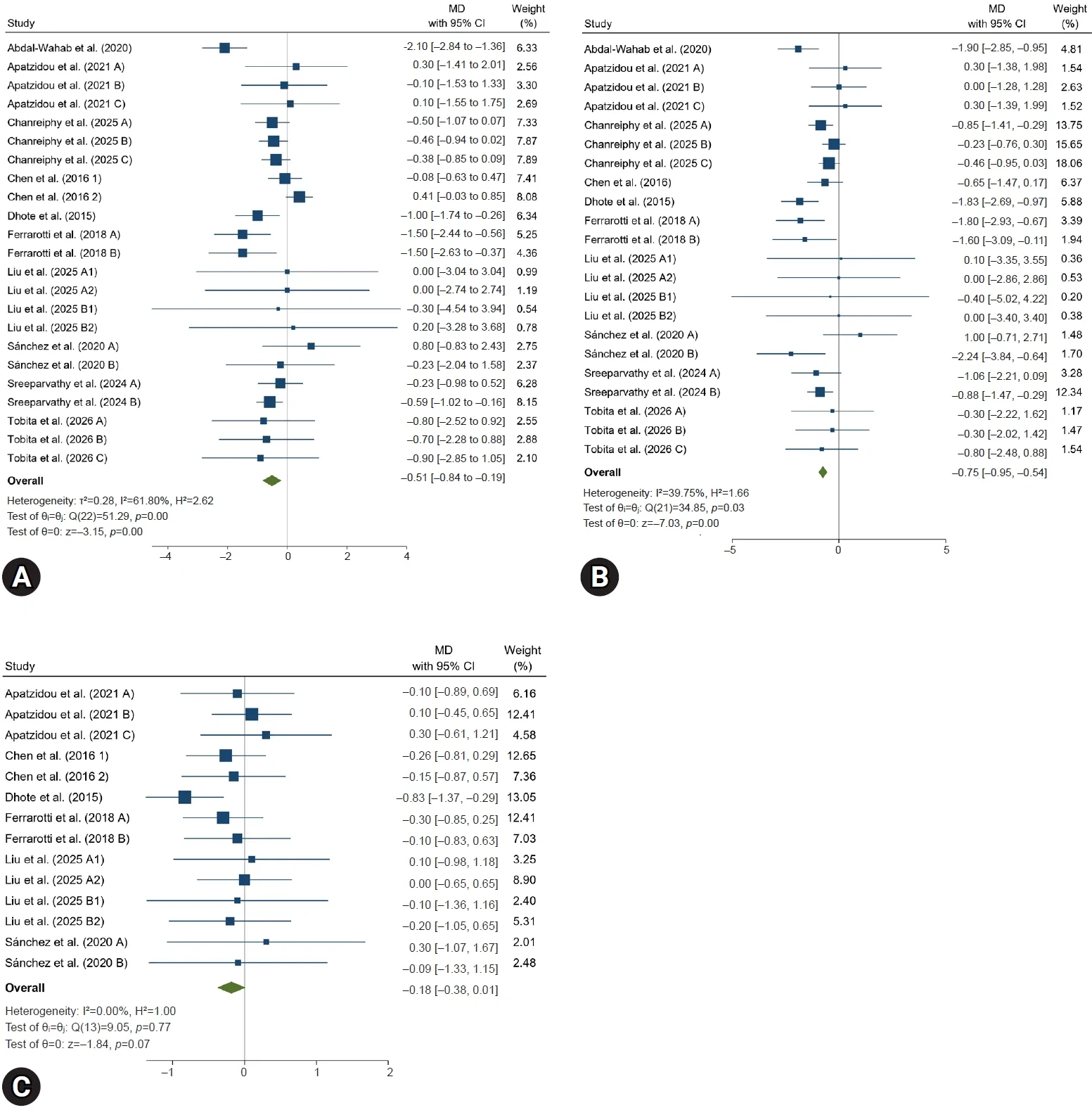
Forest plot of the efficacy of mesenchymal stem cells in (A) probing pocket depth, (B) clinical attachment level, and (C) gingival recession. MD, mean difference; CI, confidence interval.

**Fig. 4.**
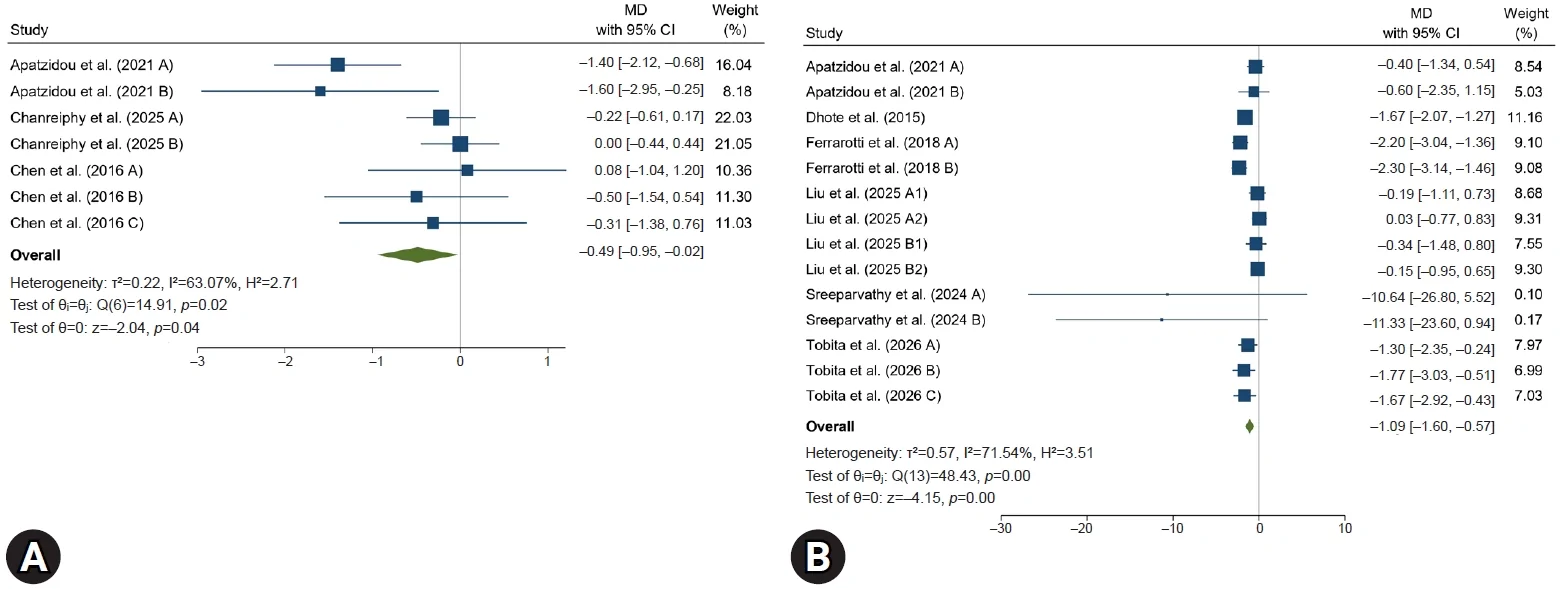
Forest plot of the efficacy of mesenchymal stem cells in (A) cementoenamel junction to the base of defect and (B) intrabony defect. MD, mean difference; CI, confidence interval.

**Fig. 5.**
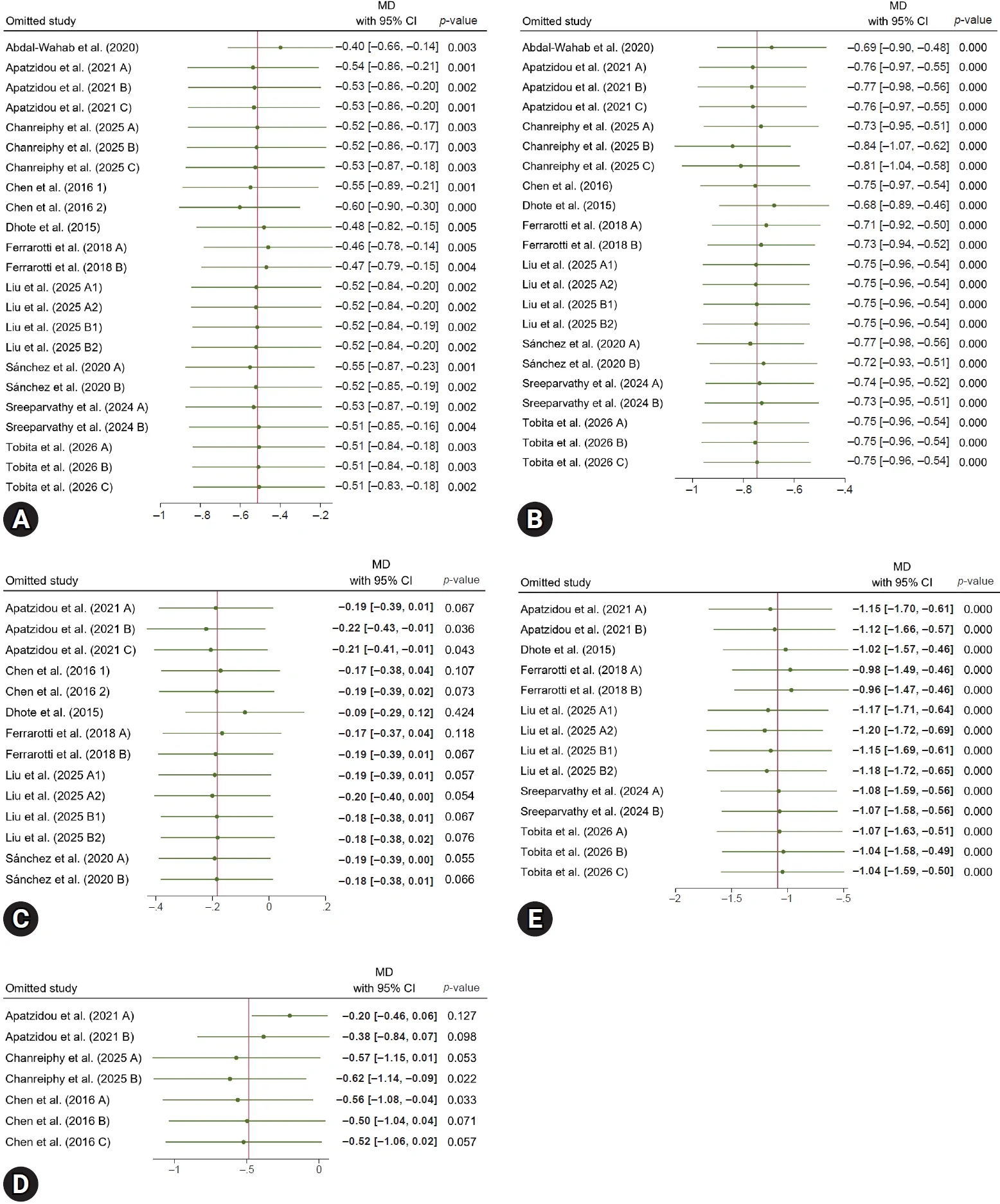
Leave-one-out sensitivity analyses for (A) probing pocket depth, (B) clinical attachment level, (C) gingival recession, (D) cementoenamel junction to the base of defect, and (E) intrabony defect. MD, mean difference; CI, confidence interval.

**Fig. 6.**
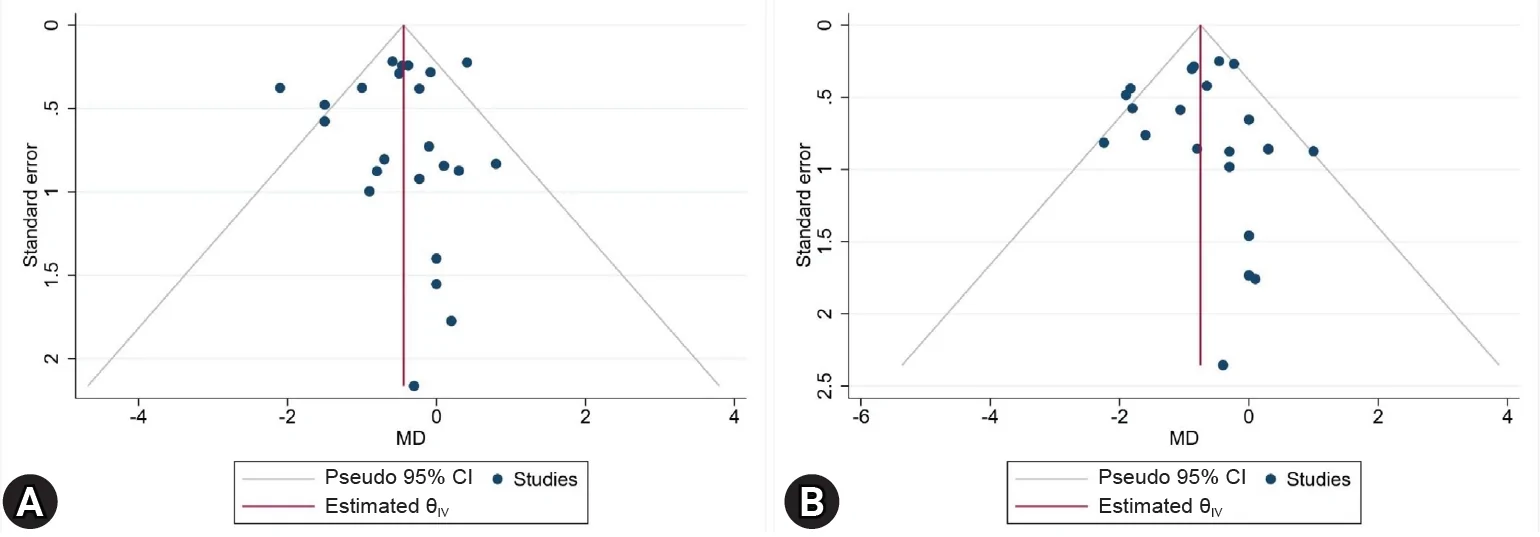
Funnel plot for (A) probing pocket depth and (B) clinical attachment level. MD, mean difference; CI, confidence interval.

**Table 1.** Study characteristics

Study	Study design	MSCs type	No. of participants^a)^	Age^b)^	Periodontal status (baseline)	Intervention (treatment group)	Intervention (control group)	Study duration (mo)
Abdal-Wahab et al. [34]	RCT	Gingival MSCs (GMSCs)	20 patients (10/10)	32–50 (42.5±4.37/43.4±3.29)	Stage III grade A/B periodontitis with 2- or 3-walled interproximal defects, transgingival sounding ≥3 mm, PPD ≥5 mm, and CAL ≥4 mm	GMSCs, β-TCP, and collagen membrane	β-TCP and collagen membrane	6
Apatzidou et al. [35]	RCT	Autologous bone marrow MSCs (BMMSCs)	19 patients (9/10)	20–68 (49.7±4.8/49.9±8.7)	Advanced periodontitis with CAL ≥6 mm, PPD ≥6 mm, 1–3 walls of intrabony defects, and intrabony component ≥3 mm	BMMSCs, autologous FPL, and collagen fleece	Autologous FPL and collagen fleece	12
Chanreiphy et al. [36]	RCT	Periodontal ligament MSCs (PDLMSCs) niche	16 patients (16 sites/16 sites)	25–50	Stage III grade B periodontitis with CAL ≥5 mm, PPD 5–8 mm, and 2- or 3-walled vertical defects of ≥3 mm	PDLMSCs niche and OFD	OFD	9
Chen et al. [37]	RCT	Autologous periodontal ligament MSCs (PDLMSCs)	30 patients (20 teeth/21 teeth)	18–65 (26.05±4.44/30.04±7.90)	Periodontitis with 2- or 3-walled vertical intrabony defect ≥3 mm	PDLMSCs, GTR, and Bio-Oss	GTR and Bio-Oss	12
Dhote et al. [38]	RCT	Human umbilical cord MSCs (UCMSCs)	14 patients (14 sites/14 sites)	20–43 (32.62±6.99)	Moderate-advanced periodontitis with CAL ≥5 mm, PPD ≥5 mm, interproximal intrabony osseous defect component ≥3 mm	UCMSCs, β-TCP, and rh-PDGF-BB	OFD	6
Ferrarotti et al. [39]	RCT	Autologous dental pulp MSCs (DPMSCs)	29 patients (15/14)	39–69 (51.9±8.4/49.4±9.3)	Chronic periodontitis with 1–3-walled vertical defect with residual PPD ≥6 mm and intrabony component ≥3 mm	Micro-graft enriched in DPMSCs and collagen sponge	Collagen sponge	12
Liu et al. [40]	RCT	Human dental pulp MSCs (DPMSCs)	96 patients (33/30/33)^c)^	18–65 (37.2±8.15/37.5±9.02/36.8±8.19)	Chronic periodontitis with PPD 4-8 mm and intrabony defect	DPMSCs injection, with a single dose or a double dose (a single dose given twice with an interval of 30 days)	Saline injection	6
Sánchez et al. [41]	RCT	Autologous periodontal ligament MSCs (PDLMSCs)	20 patients (10/10)	25–70 (48.8±10.6/57.5±7.9)	Moderate-severe chronic periodontitis or stage III-IV periodontitis with CAL ≥6 mm and 1- or 2-walled intrabony defect with radiographic intrabony component ≥4 mm	PDLMSCs and XBS	XBS and saline	12
Sreeparvathy et al. [42]	RCT	Peripheral blood MSCs (PBMSCs)	17 patients (17 sites/17 sites)	30–55 (37.7±4.4)	Stage III grade B periodontitis with PPD ≥6 mm and 3-walled bilateral intraosseous defects	PBMSCs and PRFM	PRFM	6
Tobita et al. [43]	RCT	Autologous adipose tissue MSCs (AMSCs)	15 patients (9/6)	≥20 (56.1±12.2/59.7±5.4)	Chronic periodontitis with 1–3-walled bone defects, PPD ≥5 mm, intrabony defect ≥5 mm in depth and ≥2 mm in width	AMSCs and PRP	EMD	9

MSC, mesenchymal stem cell; RCT, randomized controlled trial; GMSC, gingival mesenchymal stem cell; PPD, probing pocket depth; CAL, clinical attachment level; β-TCP, beta-tricalcium phosphate; BMMSC, bone marrow mesenchymal stem cell; FPL, fibrin/platelet lysate; PDLMSC, periodontal ligament mesenchymal stem cell; OFD, open flap debridement; GTR, guided tissue regeneration; UCMSC, umbilical cord mesenchymal stem cell; rh-PDGF-BB, recombinant human platelet-derived growth factor-BB; DPMSC, dental pulp mesenchymal stem cell; XBS, xenogeneic bone substitute; PBMSC, peripheral blood mesenchymal stem cell; PRFM, platelet-rich fibrin matrix; AMSC, adipose tissue mesenchymal stem cell; PRP, platelet-rich plasma; EMD, enamel matrix derivative.

^a)^Number of participants (treatment/control). ^b)^Age range (mean, treatment/control). ^c)^ Study with multiple arms (treatment 1/treatment 2/control).

**Table 2.** Subgroup analysis

Outcome	Subgroup	df	MD (95% CI)	*p*-value	*p*-value^a)^	Heterogeneity	
						*I*^2^ (%)	*p*-value
PPD	Overall	22	–0.51 (–0.84 to –0.19)	<0.01	NA	62	<0.01
	Study design						
	Split-mouth	5	–0.51 (–0.73 to –0.29)	<0.01	0.52	0	0.74
	Parallel-arm	14	–0.71 (–1.26 to –0.15)	0.01		43	0.06
	MSC type						
	Orally derived	13	–0.53 (–1.02 to –0.04)	0.03	0.96	76	<0.01
	Non-orally derived	8	–0.55 (–0.84 to –0.25)	<0.01		0	0.82
	Follow-up duration (mo)						
	≤6	16	–0.51 (–0.90 to –0.11)	0.01	0.92	69	<0.01
	>6	5	–0.48 (–0.88 to –0.09)	0.02		1	0.51
CAL	Overall	21	–0.75 (–0.95 to –0.54)	<0.01	NA	40	0.03
	Study design						
	Split-mouth	5	–0.71 (–0.96 to –0.45)	<0.01	0.47	57	0.04
	Parallel-arm	14	–0.89 (–1.30 to –0.47)	<0.01		38	0.07
	MSC type						
	Orally derived	12	–0.71 (–0.96 to –0.45)	<0.01	0.57	49	0.02
	Non-orally derived	8	–0.83 (–1.21 to –0.46)	<0.01		28	0.19
	Follow-up duration (mo)						
	≤6	15	–0.81 (–1.05 to –0.56)	<0.01	0.35	42	0.04
	>6	5	–0.58 (–0.98 to –0.19)	<0.01		38	0.15
CEJ-BD	Overall	6	–0.49 (–0.95 to –0.02)	0.04	NA	63	0.02
	Study design						
	Split-mouth	1	–0.12 (–0.42 to 0.17)	0.40	<0.01	0	0.46
	Parallel-arm	1	–1.44 (–2.08 to –0.81)	<0.01		0	0.80
	MSC type						
	Orally derived	4	–0.15 (–0.41 to 0.12)	0.27	<0.01	0	0.87
	Non-orally derived	1	–1.44 (–2.08 to –0.81)	<0.01		0	0.80
	Follow-up duration (mo)						
	≤6	3	–0.54 (–1.18 to 0.11)	0.10	0.88	65	0.03
	>6	2	–0.46 (–1.30 to 0.39)	0.29		61	0.08
IBD	Overall	13	–1.09 (–1.60 to –0.57)	<0.01	NA	71	<0.01
	Study design						
	Split-mouth	2	–5.22 (–12.50 to 2.07)	0.16	0.26	47	0.17
	Parallel-arm	10	–0.98 (–1.54 to –0.43)	<0.01		71	<0.01
	MSC type						
	Orally derived	5	–0.87 (–1.75 to 0.01)	0.05	0.34	84	<0.01
	Non-orally derived	7	–1.36 (–1.84 to –0.87)	<0.01		28	0.14
	Follow-up duration (mo)						
	≤6	10	–0.93 (–1.49 to –0.36)	<0.01	0.10	72	<0.01
	>6	2	–1.78 (–2.63 to –0.92)	<0.01		34	0.21

df, degrees of freedom; MD, mean difference; CI, confidence interval; PPD, probing pocket depth; NA, not applied; MSC, mesenchymal stem cell; CAL, clinical attachment level; CEJ-BD, cementoenamel junction to the base of defect; IBD, intrabony defect.

a) *p*-value for subgroup difference.

**Table 3.** Adverse events assessment

Study	Adverse events assessment
Abdal-Wahab et al. [34]	No adverse effects or complications—including allergy, abscess, or infection—were reported during the study period
Apatzidou et al. [35]	No adverse healing events were reported during the 12-month study period, including during the additional 3-year follow-up
Chanreiphy et al. [36]	No adverse events were reported
Chen et al. [37]	No adverse events or complications were reported other than swelling and moderate pain. Blood and serum tests showed clinical parameters within the normal range. In urine analysis, two patients whose teeth received MSCs therapy tested positive for albumin; no other significant changes were observed in other parameters
Dhote et al. [38]	No adverse effects, allergic reactions, infections, or patient complaints related to the graft material were reported, and the MSCs were clinically well tolerated
Ferrarotti et al. [39]	No infections were reported in any of the patients
Liu et al. [40]	MSCs injection was safe and well tolerated. Grade 1 adverse events that resolved without any treatment were reported. First, in the investigator-initiated trial, two patients in the experimental group experienced toothache and injection-site swelling, while one control patient experienced injection-site swelling. Second, in the phase I trial, two patients in the experimental group experienced gingival swelling and diarrhea, which may have been related to the injection and resolved without treatment
Sánchez et al. [41]	No adverse events were reported other than the common effects of surgery, such as mild-to-moderate pain and swelling, as well as dentin hypersensitivity in both the experimental and control groups
Sreeparvathy et al. [42]	No signs or symptoms of discomfort, infection, or unfavorable reactions were reported in any stage of the study
Tobita et al. [43]	No intervention-site adverse events were reported. The episodes of acute periodontitis in one patient and heart failure in another were not related to MSCs administration

MSC, mesenchymal stem cell.

**Table 4.** Certainty of evidence based on the GRADE (Grading of Recommendations, Assessment, Development, and Evaluation)

Domain	PPD	CAL	GR	CEJ-BD	IBD
Risk of bias	NS	NS	NS	NS	NS
Inconsistency	S	NS	NS	S	S
Indirectness	NS	NS	NS	NS	NS
Imprecision	NS	NS	S	NS	NS
Publication bias	NS	NS	NA	NA	NA
Certainty	Moderate	High	Moderate	Moderate	Moderate

PPD, probing pocket depth; CAL, clinical attachment level; GR, gingival recession; CEJ-BD, cementoenamel junction to the base of defect; IBD, intrabony defect; NS, not serious; S, serious; NA, not assessed.

## Supplementary materials

Supplementary Table 1 can be found at https://doi.org/10.12701/jyms.2026.43.42.

Supplementary Table 1. Search queries in each database
