# Supplementary material for: Therapeutic efficacy of mesenchymal stem cells in periodontal regeneration: a systematic review and meta-analysis of clinical trials
Source: J Yeungnam Med Sci. 2026 Jun 24;43:42. doi: 10.12701/jyms.2026.43.42 (PMC13681079; doi:10.12701/jyms.2026.43.42)
Supplement: Supplementary file 1 [file jyms-2026-43-42-Supplementary-Table-1.pdf]

Supplementary Table 1. Search queries in each database

Database	Queries
PubMed	((“stem cells”[MeSH Terms] OR “stem cells”[All Fields]) OR (“mesenchymal stem cells”[MeSH Terms] OR “mesenchymal stem cells”[All Fields] OR “MSC”[All Fields]) OR (“mesenchymal stromal cell” [All Fields])) AND (("periodontal"[All Fields] OR "periodontally"[All Fields] OR "periodontically"[All Fields] OR "periodontics"[MeSH Terms] OR "periodontics"[All Fields] OR "periodontic"[All Fields] OR "periodontitis"[MeSH Terms] OR "periodontitis"[All Fields] OR "periodontitides"[All Fields])) AND ("randomized controlled trial" OR “RCT" OR "clinical trial" OR "controlled clinical trial" OR “random" OR “trial" OR “placebo") NOT ("review" OR "narrative review" OR "literature review" OR "scoping review" OR "rapid review" OR "umbrella review" OR "integrative review" OR "critical review" OR "systematic review" OR "meta-analysis" OR "case study" OR "case report" OR "case series" OR "commentary" OR "editorial" OR "short communication" OR "animal study" OR "animal" OR "in vivo" OR "mouse" OR "mice" OR "murine")
Scopus	TITLE-ABS-KEY ("stem cell*" OR "mesenchymal stem cell*" OR MSC OR "mesenchymal stromal cell*") AND TITLE-ABS-KEY (periodontal OR periodontitis OR "periodontal disease" OR "periodontal regeneration") AND TITLE-ABS-KEY ("randomized controlled trial" OR RCT OR "clinical trial" OR "controlled clinical trial" OR random* OR trial OR placebo) AND NOT TITLE-ABS-KEY (review OR "narrative review" OR "literature review" OR "scoping review" OR "rapid review" OR "umbrella review" OR "integrative review" OR "critical review" OR "systematic review" OR "systematic review and meta-analysis" OR "meta-analysis" OR "case study" OR "case report" OR "case series" OR commentary OR editorial OR "short communication" OR "animal study" OR animal OR "in vivo" OR mouse OR mice OR murine) AND (LIMIT-TO (DOCTYPE , "ar")) AND (LIMIT-TO (LANGUAGE , "English")) AND (LIMIT-TO (SRCTYPE , "j"))
Web of Science	("stem cell*" OR "mesenchymal stem cell*" OR MSC OR "mesenchymal stromal cell*") (Topic) AND (periodontal OR periodontitis OR "periodontal disease" OR "periodontal regeneration") (Topic) AND ("randomized controlled trial" OR RCT OR "clinical trial" OR "controlled clinical trial" OR random* OR trial OR placebo) (Topic) NOT ("review" OR "narrative review" OR "literature review" OR "scoping review" OR "rapid review" OR "umbrella review" OR "integrative review" OR "critical review" OR "systematic review" OR "systematic review and meta-analysis" OR "meta-analysis" OR "case study" OR "case report" OR "case series" OR "commentary" OR "editorial" OR "short communication" OR "animal study" OR "animal" OR "in vivo" OR "mouse" OR "mice" OR "murine") (Topic)<br>Filter: Document types (Article), Languages (English)
Dentistry & Oral Sciences Source	XB ("stem cell*" OR "mesenchymal stem cell*" OR MSC OR "mesenchymal stromal cell*") AND XB (periodontal OR periodontitis OR "periodontal disease" OR "periodontal regeneration") AND XB ("randomized controlled trial" OR RCT OR "clinical trial" OR "controlled clinical trial" OR random* OR trial OR placebo) NOT XB (review OR "narrative review" OR "literature review" OR "scoping review" OR "rapid review" OR "umbrella review" OR "integrative review" OR "critical review" OR "systematic review" OR "systematic review and meta-analysis" OR "meta-analysis" OR "case study" OR "case report" OR "case series" OR commentary OR editorial OR "short communication" OR "animal study" OR animal OR "in vivo" OR mouse OR mice OR murine)<br>Filter: Peer-reviewed, Source types (Academic journal), Language (English)
Cochrane Library	Title Abstract Keyword: ("stem cells" OR "mesenchymal stem cells" OR MSC OR "mesenchymal stromal cells") AND (periodontal OR periodontitis OR "periodontal disease" OR "periodontal regeneration") AND ("randomized controlled trial" OR RCT OR "clinical trial" OR "controlled clinical trial" OR random OR trial OR placebo)
